# Perceived age discrimination and social isolation mediate the relationship between disasters and loneliness: results from Wave 1 of the Longitudinal Ageing Study of India

**DOI:** 10.1093/geronb/gbaf121

**Published:** 2025-06-28

**Authors:** Liat Ayalon, Sayani Das

**Affiliations:** Louis and Gabi Weisfeld School of Social Work, Bar Ilan University, Ramat Gan, Israel; Louis and Gabi Weisfeld School of Social Work, Bar Ilan University, Ramat Gan, Israel

**Keywords:** ageism, discrimination, isolation, Longitudinal Ageing Study in India

## Abstract

**Objectives:**

Disasters are an inevitable part of life, with older persons being particularly susceptible to their effects. Although social isolation (e.g., the objective lack of relationships) has an important role in making older persons more susceptible to the negative effects of disasters, less is known about the effects of disasters on social isolation and loneliness (e.g., the subjective perception of inadequate social ties). This study evaluated the mediating role of perceived age discrimination (e.g., the attribution of the experience of unfairness to one’s age) and social isolation in the relationship between disasters and loneliness.

**Methods:**

Relying on data from the Longitudinal Ageing Study of India (LASI) Wave 1 (*N* = 31,902), we applied structural equation modelling to examine the direct effect of disasters on older persons’ loneliness and their indirect effect via perceived age discrimination and social isolation.

**Results:**

While disaster exposure was directly associated with perceived age discrimination, the broader association with loneliness occurs indirectly through perceived age discrimination and social isolation.

**Discussion:**

By highlighting the negative role of disasters on older persons’ loneliness and social isolation and identifying a potential mechanism that possibly explains this link, we also highlight a window of possibilities. Addressing age discrimination at the institutional level via rules and regulations and at the interpersonal level via educational interventions and intergenerational contact can result in lower social isolation and lower levels of loneliness in older persons.

In today's world, natural disasters have become a common, undesired occurrence (Intergovernmental Panel on Climate Change, 2022). From wildfires in Spain and California, tornadoes in Florida, and typhoons and floods in the Philippines, modern life is full of environmental and climate extremes (Intergovernmental Panel on Climate Change, 2022). Likewise, worldwide, acts of terror and war have become the norm in many world regions, exacerbated by the recent Ukraine-Russia war and the Swords of Iron War (Kokori et al., 2024; Mearsheimer, 2022; Samuel, 2023). Other man-made disasters, such as car or airplane accidents or industrial calamities, are common worldwide, unrelated to a particular world region. Although these disasters affect society at large, older persons are particularly susceptible to their negative effects (Roy & Bandyopadhyay, 2023).

This study explores the effects of disasters on one’s sense of loneliness, defined as the subjective experience of having inadequate social ties (Tesch-Roemer & Huxhold, 2019). These effects are thought to be mediated by heightened levels of perceived age discrimination, characterized as perceived unfairness towards people because of their age (Rippon et al., 2014), and by heightened objective lack of social connections, known as social isolation (Tesch-Roemer & Huxhold, 2019). Although much has been written about social isolation as an amplifier of the negative effects of disasters (Eric, 2015), the link between disasters and loneliness is underexplored, though, as illustrated below, it has a strong theoretical and empirical rationale.

## Loneliness and social isolation in older persons

Loneliness, known as the subjective experience of having inadequate social ties, is distinguished from social isolation, which is the objective absence of social ties (Tesch-Roemer & Huxhold, 2019). Both the prevalence rates of loneliness and the prevalence rates of social isolation are staggering worldwide. Based on a systematic review and meta-analysis of observational studies of national representative datasets collected in 113 countries, high levels of loneliness are a substantial concern for a large portion of the population worldwide (Surkalim et al., 2022). Social isolation is also high in the general population, with 12.3% of those between the ages of 18 and 80 being identified as lacking social connections (Röhr et al., 2022).

The prevalence of loneliness and social isolation is particularly high in older age (Ayalon et al., 2012). A systematic review and meta-analysis of observational studies of high-income countries conducted between 2008 and 2020 has found that the pooled prevalence estimate of loneliness is 28.5% based on data derived from 120,000 older persons in 29 countries. The prevalence of social isolation in older persons is also high, with one in four community-dwelling older persons reporting social isolation, based on data derived from 41 studies (Teo et al., 2023). Based on a systematic review and meta-analysis, the prevalence of severe loneliness is 27.1%, the prevalence of moderate loneliness is 32.1%, whereas the prevalence of social isolation is estimated at 33.6% (Hajek et al., 2023).

There are several explanations for the high levels of loneliness and social isolation in older age (Ayalon et al., 2012). With age, older persons are likely to become increasingly more disabled and to experience a reduction in their mobility level, which prevents them from participating in social activities. Losing a spouse and/or friends is yet another source of increased loneliness and social isolation. Older persons are also likely to lose social roles and ties, due to retirement and the transition of adult children outside of the nest (Ablanque & Singson, 2022).

The high rates of loneliness and social isolation pose major public health concerns (Leigh-Hunt et al., 2017). Both loneliness and social isolation have been linked to cardiovascular events and poorer mental health outcomes (Leigh-Hunt et al., 2017). Data from the English Longitudinal Study of Ageing has shown that both loneliness and social isolation are associated with a risk for reduced activity, smoking, and a substantial number of health risk behaviors. Social isolation is also related to high blood pressure, C-reactive protein, and fibrinogen levels (Shankar et al., 2011). Both loneliness and social isolation pose risks for poor cognition (Cardona & Andrés, 2023) as well as for dementia (Guarnera et al., 2023). A systematic review that examined the effects of loneliness and social isolation on mortality found that, on average, there is a 29% increased risk for mortality attributed to social isolation, 26% attributed to loneliness, and 32% attributed to living alone (Holt-Lunstad et al., 2015).

## Disasters, loneliness, and social isolation in older persons

Older persons are particularly susceptible to both natural and man-made disasters. This has been attributed to a variety of reasons (Cornell et al., 2012). First, older persons are physiologically susceptible, given an increase in non-communicable diseases in older age, reduced physical functioning, a more impaired immune system, and higher levels of physical impairment and disability (Tuohy & Stephens, 2011). Older persons are also more socially susceptible in the face of disasters (Daddoust et al., 2018). This is partially attributed to the digital divide (Peter & Stacie, 2019), which may prevent older persons from receiving warning signs on time or from receiving immediate assistance when faced with disasters. Moreover, given their physiological susceptibility, older persons are often in need of care assistance, especially during extreme times, when evacuation is required (Runefors et al., 2021).

Social isolation is known to place older persons at a particular risk for disasters. For instance, research exploring the heatwaves in Chicago has found that older persons who were socially isolated were the most likely to die, given the limited assistance that was available to them (Eric, 2015). Similar findings were reported in the case of other natural disasters, stressing the importance of social ties as a protector in times of turmoil. Specifically, following the Great East Japan Earthquake and Tsunami in 2011, social ties in the neighborhood reduced the risk of depressive symptoms even among people who were badly affected by this catastrophic event (Sasaki et al., 2020). Likewise, in the case of man-made disasters, social support is also considered essential. Specifically, exposure to terror was linked with psychological distress via one’s sense of loneliness (Faran et al., 2024). Another study found that loneliness moderated the relationship between post-traumatic stress disorder symptoms before and after the October 7th massacre and suicide ideation (Levi-Belz et al., 2024). In this study, however, we propose that instead of viewing social isolation or loneliness as moderators of the effects of disasters on older persons, it is possible to view disasters as precipitators of social isolation and subsequently a sense of loneliness. We expect this relationship to be accounted for via perceived age discrimination.

## Ageism, perceived age discrimination, and disasters

Ageism is defined as stereotypes, prejudices, and discrimination towards individuals because of their age. Ageism can be directed towards people of any age group and can take place at the institutional, macrolevel, the interpersonal, mesolevel, and the intrapersonal, microlevel (Ayalon & Tesch-Römer, 2018). Ageism is highly prevalent, documented in multiple countries and contexts. A recent analysis based on the World Value Survey found that 1 in 2 people reports exposure to ageism (Officer et al., 2020). Likewise, 1 in 3 Europeans reports exposure to ageism (Ayalon, 2014). These rates are substantially higher than the reported exposure to sexism or racism (Ayalon, 2014). Ageism is a human rights violation that carries a substantial public health impact. It is associated with higher levels of physical impairments, a greater number of chronic conditions, worse quality of life and wellbeing, and a greater risk for mortality (E.-S. Chang et al., 2020). Given these detrimental impacts, ageism is one of the four pillars of the UN Decade of Healthy Ageing (World Health Organization, 2020).

Ageism is manifested in multiple contexts, including the healthcare system, the workforce, the legal system, the media, and access to goods and services (Ayalon & Tesch-Römer, 2018). Ageism may also manifest in the context of disasters, given its association with perceived threat (Martens et al., 2005). However, a recent study has distinguished between two types of threats. When threats are framed as resource constraints, there is a high chance for competition over scarce resources, whereas threats that are framed as external safety risks result in high levels of cooperation (Miao et al., 2024). Hence, ageism may flourish under the resource threats, but solidarity will form under external threats.

In support of this claim, it is possible to view certain man-made disasters as representing external threats, thus being responsible for bringing generations together. Under conditions of war and terror, for instance, there is some research to suggest that the entire community puts all hands-on deck and collaborates. This can be clearly seen in a recent Israeli study, which examined the experiences of ageism following the Swords of Iron War. In an online survey, 911 Israelis responded to queries about ageism during the war. As many as 42.4% of them reported a reduction or non-existence of ageism during the war, and 79.1% reported a reduction or non-existence of ageism in their own daily life during the war (Ayalon et al., 2025). A different study, also conducted in Israel, followed 56 participants before the Swords of Iron War and during the war. The study found a reduction in ageism towards younger persons, which was most notable in the case of the oldest-old (Suberry et al., under review). These studies support the notion that under external safety threats, people of different age groups join forces and become more cohesive.

The COVID-19 pandemic and the climate change discourse, on the other hand, can be considered as resource threats because they represent threats to the availability of common goods. Under such circumstances, and given prescriptive stereotypes which expect older persons not to consume too much of the greater goods of society, and to give the right of way to younger generations (North & Fiske, 2013), it is expected that ageism and intergenerational conflict will prevail. This has been the case during the pandemic (Ayalon, 2020)and is highly evident in the discourse of climate activists (Roy & Ayalon, 2022). Specifically, the climate movement is broadly seen as being fueled by younger persons (Bergmann & Ossewaarde, 2020), who feel deprived of a voice and the ability to influence via formal means (Park et al., 2021). Some of the most prominent messages promoted by the climate movement concern the responsibility of past generations for the current climate situation, which intensifies the divide between the generations and the flourishing of ageism (Ayalon et al., 2021).

In this study, we examined perceived age discrimination, the subjective sense of unfairness due to one’s age, as a proxy of the behavioral component of ageism. This proxy has been used in past research to estimate the levels of ageism in society (Ayalon, 2014). However, it is important to note its limitations as it is based on one’s subjective appreciation and therefore, may be biased by one’s emotional state (Ayalon, 2018a). Nevertheless, in the absence of a clear consensus over the measurement of objective indicators of age discrimination (Ayalon & Rothermund, 2018), researchers often rely on subjective reports (Froidevaux et al., 2025; P. de Paula Couto et al., 2023).

## Ageism, perceived age discrimination, and loneliness

Although the discourse around natural and man-made disasters has been examined from the prism of ageism and intergenerational relations, its possible relation with loneliness has not been established. Nonetheless, research has shown a link between ageism and loneliness (Shiovitz-Ezra et al., 2018). A direct pathway suggests that, because of ageism, older persons become outcasts in society, excluded from various roles and opportunities to participate in social activities, which may eventually contribute to higher levels of loneliness (Walsh et al., 2017). In the case of perceived age discrimination, older persons might become particularly sensitive to social cues and revert inwardly, thus experiencing a disruption in their social interactions and higher levels of loneliness (Lee & Bierman, 2019; Sutin et al., 2015). Older persons may also internalize negative views of themselves as being responsible for the current situation and, as a result, may be less likely to engage in social interactions and report higher levels of loneliness. In support of this claim, a study that examined the relationship between different types of care settings and loneliness found that anxiety about aging (a proxy of self-directed ageism) mediated this relationship (Ayalon, 2018b).

## This study

This study was conducted in India, a young (though rapidly aging) society, with 10.1% of its population being over the age of 60 as of 2021 (UNICEF, 2025). India is a disaster-prone country, with a humongous number of natural disasters documented over its history, resulting in a large number of casualties, including deaths, injuries, and forced migration (Kapur, 2010). In addition to numerous natural disasters, people in India also have been exposed to man-made disasters such as industrial accidents, terrorism, and riots (Kar, 2010).

Traditionally, India has been characterized by strong family support systems, with multigenerational households playing a crucial role in caregiving for older adults. However, rapid urbanization, economic migration, and shifting societal values have contributed to a gradual decline in these traditional structures (Dhillon et al., 2016). The rise of nuclear families and increased labor force participation of younger generations have led to reduced intergenerational interactions, potentially exacerbating loneliness and perceived age discrimination among older adults. A systematic review and meta-analysis of the prevalence and correlates of loneliness in India has found that the pooled prevalence of loneliness was 41% and that the burden of loneliness was higher among older persons (Hossain et al., 2020). Likewise, the rates of perceived age discrimination in India are relatively high, reported by 10.33% of the older persons over the age of 60, but rising to 11.86% among the oldest-old (Maurya et al., 2022).

Examining the association between disasters and loneliness, we assessed two hypothetically different pathways, bearing in mind the distinction between scarcity of resource threats (natural disasters) vs. external safety threats (man-made disasters):

1. Increased perceived age discrimination mediates the association between natural disasters and increased loneliness via high levels of social isolation.
2. A reduction in perceived age discrimination mediates the association between man-made disasters and reduced loneliness via lower levels of social isolation.

## Method

### Participants

The present study utilizes data from the Longitudinal Ageing Study in India (LASI) Wave I, conducted in 2017–2018. LASI is a nationally representative survey focusing on the health, economic, and social well-being of India’s aging population. The LASI Wave 1 sample was meticulously designed to ensure national representation, employing a multistage stratified area probability sampling method (details in Supplementary material Section 1). The survey covers 73,396 individuals aged 45 years and older, along with their spouses (irrespective of age). The current study included 31,902 older adults (15,340 men and 16,562 women) aged 60 years and above, who are classified as senior citizens in India.

### Outcome variable

Loneliness was assessed using a single-item measure adapted from the Centre for Epidemiological Studies Depression Scale (CES-D-10), which asked: “How often do you feel alone?.” Response options included “rarely or never (less than 1 day),” “sometimes (1 or 2 days),” “often (3 or 4 days),” and “most or all of the time e (5–7 days).” For analysis, responses of “rarely or never” and “sometimes” were coded as 0, indicating not lonely, while responses of “often” or “most/all of the time” were coded as 1, indicating frequent loneliness (Hossain et al., 2020).

### Main exposure variables

In LASI, natural disaster experience was measured using the question: “In the last five years, has your health been severely affected by disasters such as floods, landslides, extreme cold and hot weather, cyclones/typhoons, droughts, earthquakes, tsunamis, or any other natural calamities?” Human-made disaster experience was measured using the question: “In the last five years, has your health been severely affected by human-made incidents such as riots, terrorism, building collapses, fires, traffic accidents, or any other human-made incidents?” Responses for both questions were dichotomized, with 0 indicating no experience of the respective disaster type and 1 indicating an affirmative experience. A composite variable, “Disaster exposure” was created by coding a “1” if the respondent reported experiencing either a natural or human-made disaster and “0” otherwise (Muhammad et al., 2024).

### Mediators

Perceived age discrimination was assessed through a set of questions designed to capture respondents’ experiences of discrimination in daily life. Respondents were asked, “In your day-to-day life, how often have the following things happened to you?” with a list of six items (e.g., “You were treated with less courtesy or respect than other people”). Respondents who identified age as a reason for the discriminatory experiences were classified as experiencing perceived age discrimination, coded as “1,” and those who did not were classified as not experiencing perceived age discrimination, coded as “0” (Maurya et al., 2022).

As a proxy of social isolation, we used five questions adapted from the Lubben Social Network Scale ((1)“Among your family members and friends, with whom would you say you have a close relationship?,” (2) “With how many of these friends would you say you have a close relationship?,” (3) “On average, how often do you meet up with friends?,” (4) “On average, how often do you speak on the phone or by email with friends?,” and (5) “With whom do you share most of your personal matters?”). Respondents who reported no connections across all five items were classified as “yes” (coded as 1), while those who provided at least one positive response were categorized as “no” social isolation (coded as 0). The internal consistency of this measure was evaluated using Cronbach’s alpha, yielding a value of 0.616, indicating acceptable reliability.

### Covariates

The covariates included in this study age, gender, place of residence, education, employment status, economic status, caste, morbidity, and depression, were selected based on a review of existing literature on loneliness and the availability of relevant questions in the LASI questionnaire to assess these variables (Shankar & Ravi, 2024; Srivastava & Srivastava, 2023). A detailed description of the criteria used for these variables can be found in Supplementary material Section 2.

### Statistical analysis

All analyses were conducted using R statistical software (version 4.3.3) (https://www.r-project.org), incorporating individual-level survey weights to account for the complex survey design of the LASI dataset. A two-tailed *p*-value < .05 was considered statistically significant. First, descriptive statistics were used to summarize the characteristics of the study population, with weighted percentages computed to ensure representativeness. Second, bivariate analyses were conducted using chi-square tests to assess differences in loneliness prevalence across disaster exposure, ageism, social isolation, and sociodemographic characteristics. Next, we examined multicollinearity among the exploratory variables (Supplementary material Section 3) using the Variance Inflation Factor (VIF) test. The analysis showed no multicollinearity concerns, as all predictor variables had VIF values below 2. As a result, all predictor variables were included in subsequent statistical analyses. Finally, structural equation modelling (SEM) was performed using the lavaan package to examine the hypothesized mediation pathways linking disaster exposure, perceived age discrimination, social isolation, and loneliness. The model was specified with disaster exposure as the independent variable, investigating both direct and indirect effects on loneliness, mediated by perceived age discrimination and social isolation. Age group, gender, residence, employment, economic status, education, caste, morbidity status, and depression status were included as control variables to adjust for potential confounding effects. The hypothesized model was structured as follows:

#### Direct effects

(Path a): Disaster exposure → Perceived age discrimination

(Path b): Perceived age discrimination → Social isolation

(Path c): Disaster exposure → Social isolation

(Path d): Social isolation → Loneliness

(Path e): Perceived age discrimination → Loneliness

(Path f): Disaster exposure → Loneliness

#### Indirect effects

Indirect_1_: a × e (Disaster → Perceived age discrimination → Loneliness)

Indirect_2_: a × b×d (Disaster → Perceived age discrimination → Social isolation → Loneliness)

Total Indirect Effect: Indirect_1_ + Indirect_2_

Total Effect: Direct effect (f) + Total Indirect Effect

The model was estimated using Weighted Least Squares Mean and Variance Adjusted (WLSMV), an estimator appropriate for categorical and ordinal variables. Standardized path coefficients (*β*) with 95% confidence intervals (CI) were reported, and R-squared values were provided to indicate the proportion of variance explained by the model.

## Results

### Descriptive statistics

Table 1 presents the characteristics of the study participants from LASI Wave 1 (*N* = 31,902). Most participants (96.5%) experienced no exposure to disasters, while 3.5% of participants reported exposure to any disaster. A total of 14.3% experienced loneliness, while the prevalence of perceived age discrimination was 10.3%, whereas 6.8% reported social isolation. In terms of demographic distribution, 58.5% of the respondents belonged to the 60–69 age group, followed by 30.2% in the 70–79 age group, and 11.3% aged 80 years or above. Gender distribution was nearly equal, with 47.5% male and 52.5% female respondents. Regarding caste, 27.7% of participants belonged to the General category, 45.2% to Other Backward Classes, 18.9% to Scheduled Castes, and 8.1% to Scheduled Tribes. The majority (70.5%) resided in rural areas, and ∼30.8% were currently employed. Regarding economic status, 21.7% belonged to the poorest quintile, and 16.4% to the richest quintile. Educational attainment was predominantly low, with 68.0% having less than primary education. Health indicators revealed that 24.3% were multi-morbid, and 8.5% had major depression.

**Table 1.** Characteristics of study population, LASI Wave 1 (*N* = 31,902).

Variables		Sample	%	Weighted percentages
**Disaster exposure**	No	30,875	96.8	96.5
	Yes	1,027	3.2	3.5
**Natural disaster exposure**	No	31,147	97.6	97.3
	Yes	755	2.4	2.7
**Man-made disaster exposure**	No	31,568	99.0	98.9
	Yes	334	1.0	1.1
**Loneliness**	No	27,771	87.1	85.7
	Yes	3059	12.9	14.3
**Perceived age discrimination**	No	28,843	90.4	89.7
	Yes	3,059	9.6	10.3
**Social isolation**	No	29,921	93.8	93.2
	Yes	1,981	6.2	6.8
*Demographic distribution*				
**Age group**	60-69	19,211	60.2	58.5
	70-79	9,250	29.0	30.2
	≥80	3,441	10.8	11.3
**Gender**	Male	15,340	48.1	47.5
	Female	16,562	51.9	52.5
**Caste**	General	9,274	29.1	27.7
	OBC	12,137	38.0	45.2
	SC	5,157	16.2	18.9
	ST	5,334	16.7	8.1
**Residence**	Rural	21,085	66.1	70.5
	Urban	10,817	33.9	29.5
**Current employment**	No	22,529	70.6	69.2
	Yes	9,373	29.4	30.8
**Economic status**	Poorest	6,580	20.6	21.7
	Poor	6,573	20.6	21.7
	Middle	6,502	20.4	20.9
	Richer	6,259	19.6	19.2
	Richest	5,988	18.8	16.4
**Educational status**	≥Secondary	4,811	15.1	14.2
	Primary	6,065	19.0	17.8
	<Primary	21,026	65.9	68.0
**Morbidity status**	No	14,579	45.7	46.9
	Co-morbid	9,343	29.3	28.7
	Multi-morbid	7,980	25.0	24.3
**Depression status**	No	29,709	93.1	91.5
	Yes	2,193	6.9	8.5

*Note*. LASI = Longitudinal Ageing Study of India; OBC = other backward classes; SC = scheduled castes; ST = scheduled tribes.

### The prevalence of loneliness across key variables

Table 2 illustrates the prevalence of loneliness stratified by disaster exposure, perceived age discrimination, social isolation, and demographic characteristics. Participants exposed to disaster had a significantly higher prevalence of loneliness (17.5%) compared to those unexposed (14.2%) (*p* < .001). Loneliness was substantially more prevalent among those who perceived age discrimination (25.4% vs. 13.1%, *p* < .001) and those who were socially isolated (20.8% vs. 13.9%, *p* < .001). Gender differences were evident, with women experiencing higher loneliness (16.9%) than men (11.5%) (*p* < .001). Loneliness prevalence increased with advancing age, reaching 17.2% among those aged 80 and above (*p* < .001). Differences were also observed across caste groups, with the highest prevalence among participants from the Other Backward Class category (49.2%), followed by Scheduled Castes (21.3%) and General (22.9%) categories, while Scheduled Tribe participants reported the lowest prevalence (6.6%) (*p* < .001). Lower socioeconomic status (16.1%, *p* = .006) and educational status (15.2%, *p* < .001) had a higher prevalence of loneliness. Morbidity status was also significant, with higher loneliness prevalence among those with multi-morbidity (17.4%) compared to those with no morbidity (12.5%) (*p* < .001). Depression was associated with a higher prevalence of loneliness, with 27.5% of those with major depression reporting loneliness compared to 13.1% among those without (*p* < .001).

**Table 2.** Prevalence (weighted percentages) of loneliness across disaster exposure, ageism, social isolation, and background characteristics, LASI Wave 1 (*N* = 31,902).

Variables		Loneliness status		
		No	Yes	*p*-value
**Disaster exposure**	No	85.8	14.2	<.01
	Yes	82.5	17.5	
**Natural disaster exposure**	No	85.8	14.2	<.01
	Yes	82.5	17.5	
**Man-made disaster exposure**	No	85.7	14.3	<.01
	Yes	79.1	20.9	
**Perceived age discrimination**	No	86.9	13.1	<.01
	Yes	74.6	25.4	
**Social isolation**	No	86.1	13.9	<.01
	Yes	79.2	20.8	
**Age group**	60–69	85.9	14.1	<.01
	70–79	86.2	13.8	
	≥80	82.8	17.2	
**Gender**	Male	88.5	11.5	<.01
	Female	83.1	16.9	
**Caste**	General	28.5	22.9	<.01
	OBC	44.6	49.2	
	SC	18.5	21.3	
	ST	8.4	6.6	
**Residence**	Rural	85.6	14.4	.002
	Urban	85.8	14.2	
**Current employment**	No	84.7	15.3	<.01
	Yes	87.8	12.2	
**Economic status**	Poorest	83.9	16.1	.006
	Poor	87.4	12.6	
	Middle	87.3	12.7	
	Richer	84.8	15.2	
	Richest	84.7	15.3	
**Educational status**	≥Secondary	89.1	10.9	<.01
	Primary	86.3	13.7	
	<Primary	84.8	15.2	
**Morbidity status**	No	87.5	12.5	<.01
	Co-morbid	85.3	14.7	
	Multi-morbid	85.7	17.4	
**Depression status**	No	86.9	13.1	<.01
	Yes	72.5	27.5	

*Note*. LASI = Longitudinal Ageing Study of India; OBC = other backward classes; SC = scheduled castes; ST = scheduled tribes.

### Path analysis of disaster exposure and loneliness

**Figure 1.**
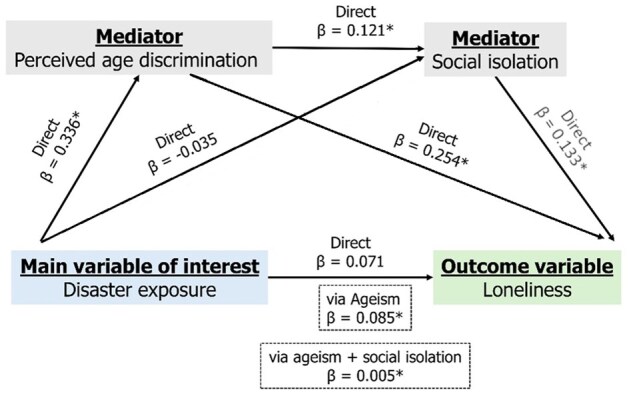
The direct and indirect pathways from disaster exposure to loneliness.

The path analysis results, presented in Table 3 and Figure 1, examine the relationship between disaster exposure and loneliness, mediated by perceived age discrimination and social isolation. We present the combined effect of disasters because the two types of disasters resulted in comparable findings, indicating similar pathways from disaster exposure to loneliness. For further clarity, a detailed breakdown of the natural and man-made disaster path analysis can be found in Supplementary material Section 4.

**Table 3.** Path analysis of disaster exposure on loneliness, via ageism and social isolation (*N* = 31,902).

Variable	Loneliness	Social isolation	Ageism
	*β* (95% CI)	*β* (95% CI)	*β* (95% CI)
*Main effects*			
**Disaster exposure**			
**No**			
**Yes**	0.071 (−0.027, 0.170)	−0.035 (−0.159, 0.090)	0.336 (0.242, 0.429)*
Perceived age discrimination			
**No**			
**Yes**	0.254 (0.224, 0.284)*	0.121 (0.085, 0.157)*	
Social isolation			
**No**			
**Yes**	0.133 (0.099, 0.166)*		
**R square**	0.122	0.035	0.039
*Indirect effect*			
**Any Disaster → Loneliness (via perceived age discrimination)**	0.085 (0.059, 0.111)*		
**Any Disaster → Loneliness (via perceived age discrimination and social isolation)**	0.005 (0.003, 0.008)*		
**Total indirect**	0.091 (0.063, 0.118)*		
**Total effect**	0.162 (0.065, 0.259)*		

*Note*. CI = confidence interval.

All effects are adjusted for age group, gender, caste, residence, employment, economic status, education, morbid status, and depression status.

* *p* < .001 significance level.

Disaster exposure was positively associated with perceived age discrimination (*β*  =  0.336, 95% CI: 0.242, 0.429, *p* < .01), but not significantly related to loneliness (*β*  =  0.071, 95% CI: −0.027, 0.170) or social isolation (*β* = −0.035, 95% CI: −0.159, 0.090). Perceived age discrimination, in turn, had a robust positive effect on loneliness (*β*  =  0.254, 95% CI: 0.224, 0.284, *p* < .001) and a positive effect on social isolation (*β*  =  0.121, 95% CI: 0.085, 0.1657, *p* < .001). Social isolation was positively associated with loneliness (*β*  = 0.133, 95% CI: 0.099, 0.166, *p* < .001). These results suggest that while disaster exposure was directly associated with perceived age discrimination, the broader association with loneliness occurs indirectly through perceived age discrimination and social isolation. Specifically, disaster exposure contributed to increased loneliness through ageism (*β*  =  0.085, 95% CI: 0.059, 0.111, *p* < .001) and both perceived age discrimination and social isolation (*β*  =  0.005, 95% CI: 0.003, 0.008, *p* < .001). The total indirect effect was 0.091 (95% CI: 0.063, 0.118, *p* < .001), suggesting that the pathway through perceived age discrimination and social isolation is significantly associated with higher levels of loneliness following disaster exposure. All effects were adjusted for sociodemographic and health covariates, including age group, gender, caste, residence, employment, economic status, education, morbidity status, and depression status.

## Discussion

This innovative study had several aims. First, we attempted to differentiate between the two types of disasters as possibly reflecting different types of threat (Miao et al., 2024). Namely, natural vs. man-made disasters, and examine their differential association with loneliness. Second, to account for the association between disasters and loneliness, we evaluated the role of perceived age discrimination and social isolation as possible mediators. Our research hypotheses were partially supported, suggesting a link between disasters (either natural or man-made) and perceived age discrimination, which either directly or via social isolation is associated with loneliness.

India’s disaster management framework has made strides in addressing vulnerable populations, yet older persons remain an often-overlooked group in disaster preparedness and response policies. The National Policy for Older Persons (1999) and the Maintenance and Welfare of Parents and Senior Citizens Act (2007) emphasize elder welfare, but they do not specifically integrate disaster resilience strategies for older persons. Furthermore, while the Disaster Management Act (2005) mandates a comprehensive response framework, it lacks targeted interventions for the older adults, who may face mobility constraints, pre-existing health conditions, and digital exclusion in emergency communication.

The present findings are important because they identified underexplored consequences of disasters, namely loneliness, social isolation, and perceived age discrimination. Moreover, the study explores a possible pathway to account for the association between disasters and loneliness by highlighting a relationship between perceived age discrimination following disaster exposure and higher levels of social isolation and loneliness.

This association between perceived age discrimination and the disruption of social ties could possibly be explained either by an increase in self-directed ageism, following the internalization of negative societal attitudes towards older persons, which in turn results in social isolation. Alternatively, perceived age discrimination might reflect exclusionary practices, which result in the disruption of social ties by contributing to higher levels of social isolation and loneliness (Shiovitz-Ezra et al., 2018). Such explanatory pathways between perceived age discrimination and loneliness were previously proposed in a large-scale study based on the Health and Retirement Study in the United States (Sutin et al., 2015). There is plenty of research to show the negative effects of loneliness and perceived age discrimination on older persons’ health, mental health, and even mortality (Beutel et al., 2017; E. S. Chang et al., 2020; Courtin & Knapp, 2017). Therefore, highlighting the connection between the two and identifying factors that explain their etiology is important.

In contrast to our expectations, there was no difference between natural and man-made disasters. This can possibly be accounted for by the fact that many different types of disasters (and therefore, threats) can be characterized as man-made disasters. Whereas terror and wars likely pose an external threat, accidents may not. Moreover, although, consistent with the LASI survey terminology, the term natural disasters was used in this study, we believe that this term is a misnomer. We claim that natural hazards become (un(natural disasters due to the failure of institutions to protect the most vulnerable (Puttick et al., 2018). This makes the distinction between threats due to scarcity of resources vs. external threats to one’s safety almost impossible. Future research will benefit from collecting more refined data concerning disasters.

Our findings demonstrate a fully mediated link between disasters and loneliness, either directly via perceived age discrimination or via perceived age discrimination followed by social isolation. Exposure to disasters represents an imminent threat to one’s well-being, health, and sense of safety (Benevolenza & DeRigne, 2019; Daddoust et al., 2018; Parker et al., 2016). Under such circumstances of extreme threat, social relations are essential (Cherry et al., 2015; Sasaki et al., 2020). Yet, extreme threats may also result in discriminatory practices and conflicts between different segments of society, including direct ageist practices towards older persons as was recently seen in the case of the pandemic (Ayalon, 2020).

The study points to a possible mechanism responsible for the association between disasters, social isolation, and loneliness, namely, perceived age discrimination. Ageism, which also incorporates a behavioral component assessed in this study, is often represented in the form of intergenerational tension and conflict (North & Fiske, 2012). It has been shown to intensify at times of scarce resources, which can be brought by the changing climate, but also by the pandemic or other situations which pose a threat to the shared goods of society (Ayalon, 2020; Elliott, 2022; Gardiner, 2006; Murphy, 2021; Roy & Ayalon, 2022). Our findings show that regardless of disaster type, perceived age discrimination is an important mediator, which possibly accounts for the relationship between disasters, social isolation, and loneliness.

Currently, two interventions have shown evidence in reducing ageism, namely education and intergenerational contact (Apriceno & Levy, 2023; Burnes et al., 2019). Although educational interventions to reduce ageism have focused mainly on improving knowledge of ageism, aging, and older age, it is possible that educational interventions around areas of threat and divide between the generations will also result in reduced ageism. For instance, providing educational information about the importance of pro-environmental behaviors and climate activism can result not only in protecting the climate and contributing to the climate change movement but also in bringing generations together and possibly reducing ageism by uniting intergenerational efforts. This, in turn, can result in a reduced sense of social isolation and loneliness. It is important to note, however, that current interventions have been primarily successful in reducing ageist attitudes and stereotypes, but not in addressing discrimination towards older persons (Apriceno & Levy, 2023; Burnes et al., 2019), which was examined in this study.

Our study points to underexplored social outcomes associated with disasters, namely an increase in perceived age discrimination, followed by an indirect increase in social isolation and a decrease in loneliness. We show that following exposure to disaster(s), when older persons are most in need of social support, they experience a disruption in their social ties, with objective and subjective ramifications in the form of increased social isolation and loneliness. These findings are particularly concerning, given past research which has highlighted the social vulnerability of older persons in the face of extreme situations (Eric, 2015).

When reviewing the findings, it is important to point out their limitations. First, as already noted, our ability to differentiate between threats due to scarcity of resources and threats due to external forces was limited, given the arbitrary division between natural vs. man-made disasters in the present study. Second, the study consisted of a single wave of data collection, thus, we cannot differentiate between cause and effect. Third, it is very possible that the association between disasters and loneliness is mediated not only by perceived age discrimination but also by self-directed ageism. When people perceive their own aging as putting them in an inferior position, this is likely to impair their social relations and contribute to their sense of loneliness. However, this variable is not measured in the LASI data. Further research will benefit from examining its link with loneliness. Likewise, other factors such as forced migration or the degradation of the environment could also account for the increase in loneliness found in the present study. These can also be examined in future research. Moreover, we assessed perceived age discrimination, rather than objective indicators of age discrimination. As already noted, this has its limitations, given the reliance on one’s subjective reports (Ayalon, 2018b), but is considered a common practice in the absence of objective indicators (Ayalon & Rothermund, 2018). In addition, the reliance on a single item to assess loneliness represents a less-than-ideal solution, which is often employed in large-scale surveys to cover multiple domains, relying on a minimal number of items per domain. Nonetheless, a recent study comparing various measures of loneliness suggested that even the use of a single item of loneliness is psychometrically ­justified (Mund et al., 2023).

Despite its limitations, this study highlights the underexplored, under-recognized effects of disasters on older persons. Both loneliness and perceived age discrimination represent public health threats, which are well-known for impairing older persons’ wellbeing and health (Beutel et al., 2017; E. S. Chang et al., 2020; Courtin & Knapp, 2017). By highlighting the negative role of disasters on older persons’ loneliness and social isolation and identifying a potential mechanism that possibly explains this link, we also highlight a window of opportunity. Addressing ageism (including age discrimination) at the institutional level via rules and regulations and at the interpersonal level via educational interventions and intergenerational contact can result in lower social isolation and lower levels of loneliness in older persons. As our study shows that disasters often precipitate higher levels of perceived age discrimination, it is possible that educational interventions and intergenerational contact will form around the main sources of disaster and threat. Such interventions should be geared towards bringing together people of different generations around a common goal and reducing ageism.

A recent review has shown that only 18 of the US States specifically address older persons in their climate policy (Carlson et al., 2024). This can possibly be considered an act of age discrimination as States fail to acknowledge the susceptibility of older persons. Likewise, we know from research concerning the use of digital technologies that some older persons find it challenging to navigate new technologies, partially because of self-directed ageist stereotypes and partially because of ageism directed by those responsible for mediating new technologies on their behalf (Köttl et al., 2021). As digital technology has become the norm in disaster mitigation, adaptation, and preparedness (Niyazi & Behnamian, 2023), it is important to ensure that older persons have adequate tools to negotiate new technological innovations.

## Supplementary Material

gbaf121_Supplementary_Data

## Data Availability

The data that support the findings of this study are publicly available through the International Institute for Population Sciences (IIPS), Mumbai. The data can be accessed via the official repository at: https://www.iipsindia.ac.in/content/LASI-data
