# Supplementary material for: Perceived age discrimination and social isolation mediate the relationship between disasters and loneliness: results from Wave 1 of the Longitudinal Ageing Study of India
Source: J Gerontol B Psychol Sci Soc Sci. 2025 Jun 28;80(8):gbaf121. doi: 10.1093/geronb/gbaf121 (PMC12313021; doi:10.1093/geronb/gbaf121)
Supplement: gbaf121_Supplementary_Data [file gbaf121_supplementary_data.zip › JGSS suppl Ayalon & Das.docx]

***The Journals of Gerontology, Series B: Psychological Sciences and Social Sciences* Supplementary Material: Ayalon & Das. Perceived age discrimination and social isolation mediate the relationship between disasters and loneliness: Results from Wave 1 of the Longitudinal Ageing Study of India.**

**Section 1: Multistage Sampling Design of LASI Wave I^[[1]](#footnote-1)^**

- 1. Rural Sampling (Three-Stage Design)

1. First Stage (Selection of PSUs): Primary Sampling Units (PSUs) were identified as sub-districts, commonly referred to as Tehsils or Talukas. These units were selected systematically to represent the diverse rural landscape across all states and union territories in India.
2. Second Stage (Selection of SSUs): From each selected PSU, villages were chosen as Secondary Sampling Units (SSUs). The selection was based on population size and geographic distribution to ensure inclusiveness and diversity.
3. Third Stage (Selection of Households): Within each selected village, households were randomly selected. This stage ensured that a broad spectrum of socio-economic conditions was represented within the rural sample.
   1. Urban Sampling (Four-Stage Design)
4. First Stage (Selection of PSUs): Similar to the rural approach, urban PSUs were defined as sub-districts (Tehsils/Talukas) and selected to ensure comprehensive geographic coverage.
5. Second Stage (Selection of SSUs): Wards within the selected urban PSUs served as SSUs. The selection process considered the demographic diversity of urban areas.
6. Third Stage (Selection of CEBs): Given the larger size of urban wards, one Census Enumeration Block (CEB) was randomly selected from each ward to streamline the household listing process.
7. Fourth Stage (Selection of Households): Households were then randomly selected from the chosen CEBs, ensuring adequate representation of urban demographic diversity.

LASI employed Computer-Assisted Personal Interviewing (CAPI) technology for data collection, enabling direct digital entry of responses during face-to-face interviews, thereby minimizing data entry errors. Field teams used pre-loaded laptops to record responses in real time, reducing the need for post-survey data transcription. To ensure data quality, LASI implemented interviewer training, pilot testing, and quality control measures such as random spot checks, systematic data reviews, and validation checks for range, logic, and consistency. Post-collection procedures included data verification, cross-referencing with external sources, follow-up interviews, and statistical checks to enhance reliability and accuracy.

**Section 2: List of covariates**

The following are the variables and their coding procedure:

1. Age group: Age group was classified into three categories: 60–69 years, 70–79 years, and 80 years and above.
2. Gender: Gender was recorded as male or female.
3. Caste: Classified into four groups as Scheduled Tribes (ST), Scheduled Castes (SC), Other Backward Classes (OBC), and General (none of the above). Caste holds significant socio-cultural and political importance in India. The Government of India officially classifies the population into these four major social groups: Scheduled Tribes (ST), Scheduled Castes (SC), Other Backward Classes (OBC), and the General category (which includes individuals not identified under the previous three categories). This categorization, which reflects long-standing social hierarchies and inequities, is also adopted in the LASI Wave 1 dataset. Historically marginalized communities, such as the ST and SC populations, have faced systemic discrimination and limited access to social, educational, and economic opportunities. OBC communities, though relatively better positioned, continue to encounter structural disadvantages compared to the General category.
4. Place of residence: Place of residence was categorized as urban or rural.
5. Education status: Education status was divided into four groups: less than primary education, primary completed, and secondary education or higher.
6. Employment status: Employment status was classified as currently working or non-working.
7. Economic factors: Economic status was assessed through Monthly Per Capita Expenditure (MPCE) variable, where MPCE denotes total monthly household consumption expenditure divided by household size. Includes households’ per capita spending on food and non-food items, including health, education, utilities, etc. Participants were then classified into quintiles based on MPCE, ranging from the poorest to the richest.
8. Morbidity: Morbidity was assessed through self-reported responses to the question: “Have you ever been diagnosed with any of the following chronic conditions?” The conditions included hypertension, diabetes, cancer, chronic lung disease, chronic heart disease, stroke, arthritis, neurological disorders, and high cholesterol. Based on the number of reported conditions, morbidity was categorized into three groups: no morbidity (no diagnosed conditions), co-morbidity (a single diagnosed condition), and multimorbidity (two or more diagnosed conditions).
9. Depression: Major depression among older adults was assessed using the Composite International Diagnostic Interview Short Form (CIDI-SF), which consists of a series of screening and symptom-related questions. The screening process begins with the question: “During the last 12 months, was there ever a time when you felt sad, blue, or depressed for two weeks or more in a row?” with response options: Yes (coded as 1) and No (coded as 0). If the response is Yes, follow-up questions assess the duration and frequency of these feelings. For instance, participants are asked: “Please think of the two-week period during the last 12 months when these feelings were at their worst. During that time, did the feelings of being sad, blue, or depressed usually last all day long, most of the day, about half the day, or less than half the day?” with response options: (a) All day long, (b) Most of the day, (c) About half the day, and (d) Less than half the day. Another question follows: “During those two weeks, did you feel this way every day, almost every day, or less often than that?” with response options: (a) Every day, (b) Almost every day, and (c) Less often. Both questions are dichotomized, with responses (a) and (b) coded as 1. Further questions assess symptoms such as loss of interest, fatigue, appetite changes, concentration difficulties, feelings of worthlessness, thoughts of death, and sleep disturbances. Each symptom is coded as 1 if present and 0 if absent. The total symptom score ranges from 0 to 10, with a diagnosis of depression assigned if the respondent reports three or more symptoms. Depression is coded as 0 for ‘not diagnosed with depression’ and 1 for diagnosed with ‘depression.’ The CIDI-SF demonstrated high reliability in this sample, with a Cronbach’s alpha of 0.954.

**Section 3: Result of multicollinearity test**

| **Supplementary Table 1. Result of multicollinearity test (N=31902)** | | | |
| --- | --- | --- | --- |
|  | Tolerance value | VIF value | Remarks |
| Disaster exposure | 0.993 | 1.007 | No multicollinearity |
| Perceived age discrimination | 0.985 | 1.015 | No multicollinearity |
| Social isolation | 0.995 | 1.005 | No multicollinearity |
| Age group | 0.930 | 1.075 | No multicollinearity |
| Gender | 0.924 | 1.082 | No multicollinearity |
| Caste | 0.914 | 1.094 | No multicollinearity |
| Residence | 0.914 | 1.094 | No multicollinearity |
| Current employment | 0.825 | 1.212 | No multicollinearity |
| Economic status | 0.944 | 1.059 | No multicollinearity |
| Educational status | 0.726 | 1.377 | No multicollinearity |
| Morbidity status | 0.891 | 1.122 | No multicollinearity |
| Depression status | 0.982 | 1.018 | No multicollinearity |

**Section 4: Disaster exposure and loneliness: Natural and man-made disaster path analysis**

| **Supplementary Table 2a: Path analysis of natural disaster exposure on loneliness, via perceived age discrimination and social isolation (N=31,902)** | | | | |
| --- | --- | --- | --- | --- |
| **Main effects**,  β (95% CI) | | Loneliness | Social isolation | **Perceived age discrimination** |
| Natural disaster | No® |  |  |  |
|  | Yes | 0.103  (-0.008, 0.214) | -0.035  (-0.179, 0.109) | 0.333  (0.226, 0.441)* |
| **Perceived age discrimination** | No® |  |  |  |
|  | Yes | 0.254  (0.224, 0.284)* | 0.121  (0.085, 0.157)* |  |
| Social isolation | No® |  |  |  |
|  | Yes | 0.132  (0.099, 0.166)* |  |  |
| **R square** |  | 0.121 | 0.035 | 0.039 |
| **Indirect effect**, β (95% CI) | | | | |
| Nat. disaster → Lon. (via **perceived age discrimination** ) | | | 0.085 (0.055, 0.114)* | |
| Nat. disaster → Lon. (via **perceived age discrimination** and social isolation) | | | 0.005 (0.003, 0.008)* | |
| Total indirect | | | 0.090 (0.059, 0.121)* | |
| Total effect | | | 0.193 (0.091, 0.303)* | |
| **Supplementary Table 2b: Path analysis of man-made disaster exposure on loneliness, via perceived age discrimination and social isolation (N=31,902)** | | | | |
| **Main effects**,  β (95% CI) | | Loneliness | Social isolation | **Perceived age discrimination** |
| Man-made disaster | No® |  |  |  |
|  | Yes | 0.081  (-0.091, 0.253) | 0.006  (-0.204, 0.217) | 0.327  (0.164, 0.489)* |
| **Perceived age discrimination** | No® |  |  |  |
|  | Yes | 0.255  (0.225, 0.285)* | 0.121  (0.085, 0157)* |  |
| Social isolation | No® |  |  |  |
|  | Yes | 0.132  (0.099, 0.166)* |  |  |
| **R square** |  | 0.121 | 0.035 | 0.037 |
| **Indirect effect**, β (95% CI) | | | | |
| MM disaster → Lon. (via **perceived age discrimination**) | | | 0.083 (0.041, 0.126)* | |
| MM disaster → Lon. (via **perceived age discrimination** and social isolation) | | | 0.005 (0.002, 0.008)* | |
| Total indirect | | | 0.088 (0.043, 0.134)* | |
| Total effect | | | 0.169 (0.010, 0.338)* | |

**Note:** All effects are adjusted for age group, gender, residence, employment, economic status, education, caste, morbid status, and depression status. *p < 0.001 significance level.

1. <https://www.iipsindia.ac.in/lasi> (Access on 20.03.2025) [↑](#footnote-ref-1)
